# Multi-Stage Cortical Plasticity Induced by Visual Contrast Learning

**DOI:** 10.3389/fnins.2020.555701

**Published:** 2020-12-21

**Authors:** Jie Xi, Pan Zhang, Wu-Li Jia, Nihong Chen, Jia Yang, Ge-Tong Wang, Yun Dai, Yudong Zhang, Chang-Bing Huang

**Affiliations:** ^1^CAS Key Laboratory of Behavioral Science, Institute of Psychology, Chinese Academy of Sciences, Beijing, China; ^2^Department of Psychology, University of Chinese Academy of Sciences, Beijing, China; ^3^Center for Neural Science, New York University, New York, NY, United States; ^4^School of Education Science, Huaiyin Normal University, Huaian, China; ^5^Department of Psychology, School of Social Sciences, Tsinghua University, Beijing, China; ^6^THU-IDG/McGovern Institute for Brain Research, Beijing, China; ^7^Institute of Optics and Electronics, Chinese Academy of Sciences, Chengdu, China; ^8^The Key Laboratory on Adaptive Optics, Chinese Academy of Sciences, Chengdu, China

**Keywords:** contrast gain, ERP, latency, perceptual learning, response gain

## Abstract

Perceptual learning, the improved sensitivity via repetitive practice, is a universal phenomenon in vision and its neural mechanisms remain controversial. A central question is which stage of processing is changed after training. To answer this question, we measured the contrast response functions and electroencephalography (EEG) before and after ten daily sessions of contrast detection training. Behavioral results showed that training substantially improved visual acuity and contrast sensitivity. The learning effect was significant at the trained condition and partially transferred to control conditions. Event-related potential (ERP) results showed that training reduced the latency in both early and late ERPs at the trained condition. Specifically, contrast-gain-related changes were observed in the latency of P1, N1-P2 complex, and N2, which reflects neural changes across the early, middle, and high-level sensory stages. Meanwhile, response-gain-related changes were found in the latency of N2, which indicates stimulus-independent effect in higher-level stages. In sum, our findings indicate that learning leads to changes across different processing stages and the extent of learning and transfer may depend on the specific stage of information processing.

## Introduction

Visual perceptual learning (VPL) is a long-term performance improvement in visual tasks as a result of training or experience (Petrov et al., 2005; Sagi, 2011; Deveau et al., 2013; Dosher et al., 2013; Watanabe and Sasaki, 2015). The observed specificity to the trained stimulus, task, or retinal location in psychophysical studies has been generally taken as evidence for neural plasticity in early visual cortex (Karni and Sagi, 1991; Gilbert, 1994; Schoups et al., 1995; Watanabe et al., 2002; Chen and Fang, 2011; Crist et al., 2014). Alternatively, Mollon and Danilova (1996) hypothesized that learning occurs at a more central site but still predicts orientation and location specificity of learning. Models like improved readout or reweighting of representation neurons (e.g., V1) (Poggio et al., 1992; Dorsher and Lu, 1998) and the involvement of high-level processes beyond the visual cortex (Li W. et al., 2008) have been proposed in the last decades and received support from psychophysical (Liu, 1999; Liu and Weinshall, 2000; Xiao et al., 2008; Zhang et al., 2010), neurophysiological (Law and Gold, 2008), and brain imaging studies (Chen et al., 2015, 2017).

However, there is a growing consensus that perceptual learning involves neural processing in multiple brain regions. The reverse hierarchy theory proposed that learning back-propagate from higher to lower visual areas, providing predictive signals to lower-levels and learning site(s) depending on the task difficulty (Friston, 2003; Ahissar and Hochstein, 2004). Indeed, learning a simple task may involve a broad set of brain systems undergoing changes in sensory representations, read-out weights, decision rules, attention and feedback processes as well as sensorimotor changes (Maniglia and Seitz, 2018). The distribution of changes across the neural system may depend upon the physical stimuli as well as the training task. A similar two-stage model suggests that feature-based plasticity occurs in the early sensory processing stages, while task-based plasticity occurs in higher-level processing stages (Sasaki et al., 2013; Shibata et al., 2014, 2016).

Human electrophysiological studies can provide unique contributions to the question regarding learning stages, given different components of ERP reflected processing in different stages along the visual hierarchy (Voorhis and Hillyard, 1977; Luck et al., 2000; Fabiani et al., 2007). Modulations in both the early and late ERP components have been found in different perceptual training studies, ranging from early C1/P1 (Pourtois et al., 2008; Bao et al., 2010; Zhang et al., 2015) to enhancement in N1, P2 (Song et al., 2005; Shoji and Skrandies, 2006; Qu et al., 2010; Wang et al., 2010; Zhang et al., 2013), and later N2 and P3 components (Skrandies and Fahle, 1994; Wang et al., 2010; Hamamé et al., 2011). However, few studies have compared the contribution of early and late ERP components to perceptual learning within a unified theoretical framework.

**FIGURE 1:**
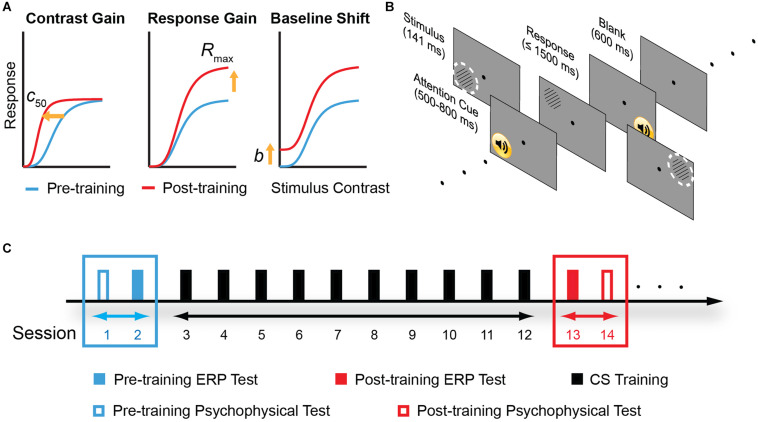
Model predictions, experimental stimuli and protocol. **(A)** Three different mechanisms in the sensory gain model that predict different pattern of contrast response function (CRF) changes following perceptual learning. From left to right: change in contrast gain, multiplicative response gain, or baseline shift. *c*_50_: the stimulus contrast that evokes half of the neuron’s maximal response. *R*_*max*_: maximal response to stimuli. *b*: baseline activity. **(B)** A typical trial procedure. Each trial started with an attention cue (500–800 ms). Stimulus was presented for 114 ms, and subjects were asked to report grating orientation within 1,500 ms. After response or 1,500 ms, a blank screen was presented for 600 ms and next trial started afterward. Training was performed in the upper left visual field location relative to the fixation dot. The dashed, white circles indicate two control locations: the upper right and the lower left visual field location. To ensure task compliance, subjects were asked to focus on the central fixation dot and press corresponding key when the black dot changed to “x” or “o” (with 5% probability each). **(C)** Experimental design. Participants were instructed to practice contrast detection tasks for ten consecutive days. Pre- and post-training psychophysical measurements covered contrast sensitivity function and visual acuity. ERP tests were conducted before and after contrast sensitivity training in different days to examine learning-induced changes in neural processing.

In this study, we tested the multi-stage hypothesis of perceptual learning. Importantly, we measured ERP with quantitative modeling based on contrast response function (CRF) measurements. In this model, the facilitation of perceptual sensitivity induced by perceptual learning could be accounted for by three possible mechanisms – increased contrast gain, increased response gain, or additive baseline shift (Figure 1A). The contrast-gain change model predicts that changes in the ERP components interact with contrast level and lead to a leftward shift in the CRF, i.e., shifting the most sensitive operating range of the system toward lower contrast while the saturation points of the CRF remain fixed. The response-gain model predicts that learning leads to a constant multiplicative change in the ERP components irrespective of the contrast level, signifying by both slope and asymptotic changes of the CRF. The baseline shift model predicts that learning leads to an overall upward, additive gain of the ERP response. We also tested the psychophysical and electrophysiological transfer effect of learning in four control conditions that varied in spatial frequency, retinal location, and eye of origin.

## Materials and Methods

### Subjects

Twenty subjects (23.8 ± 3.8 years, 12 males) participated in the study. All subjects were right-handed and had no psychiatric or neurological disorders, naïve to the task, and of normal or corrected-to-normal vision. All subjects received basic subsidies for their participation and additional bonus if they complete the whole experiments seriously. The study was approved by the Ethical Review Committee of Institute of Psychology, Chinese Academy of Sciences, and informed consent was obtained from each subject.

### Apparatus and Stimuli

The experiments were controlled by a desktop computer running Matlab programs (Mathworks, Natick, Massachusetts) and PsychToolBox3 (Brainard, 1997; Pelli, 1997). The stimuli were presented on a gamma-corrected SONY G220 CRT monitor with a spatial resolution of 1600 × 1200 pixels, a refresh rate of 85 Hz, and a mean luminance of 28.7 cd/m^2^. A special circuit combined two 8-bit output channels of the graphics card to produce 14-bit gray-level resolution (Li et al., 2003). Subjects viewed the stimuli monocularly with head on a chin rest.

Stimuli were circular sinusoidal gratings, subtending 2° at a distance of 1.38 meters, whose edges were smoothed to the background with a half-Gaussian ramp (σ = 0.31°) to minimize edge effects. The stimulus centered at 5° away from the fixation point in the upper left (trained location), upper right, or lower left location, depending on the test conditions (Figure 1B). The stimulus orientation was 45 or 135° relative to horizontal. Stimulus position was jittered slightly (0 – 0.5°) from trial to trial.

### Experimental Design

The experiment consisted of pre-training assessment, training, and post-training re-assessment (Figure 1C). Training consisted of 10 sessions; each session was composed of seven blocks of 80 trials and lasted about 30–40 min. In both pre- and post-training assessments, contrast sensitivity function (CSF), visual acuity, and ERP recordings were measured in both eyes. CSF and visual acuity were measured on the first and last day of assessment, taking up to a total of ∼ 40 min. The ERP recordings were performed in the second and the day before the last day of assessment, taking up to a total of 3.5 h (including preparation of ERP recording, data acquisition, and voluntary breaks).

### Tasks

Subjects performed a peripheral orientation discrimination task during all the CSF measurements, training, and EEG sessions (Figure 1B). Each trial started with a 500–800 ms blank (randomly jittered in time to minimize anticipation and was signaled by a brief tone) and was followed by a grating of 141 ms. Subjects indicated the orientation of the grating by a keypress within 1,500 ms. During training, a brief tone followed each correct response; during pre- and post-tests, a brief tone followed each response regardless of its accuracy. The next trial started after a 600 ms blank. Subjects were instructed to maintain fixation on a black dot at the center of the display. To ensure central fixation, the dot was randomly changed to letter “x” or “o” at a probability of 0.1, and subjects were asked to indicate the change with keypress, i.e., central task.

### Pre- and Post-training Psychophysical Assessments

Visual acuity was measured with the Chinese Tumbling E Chart (Mou, 1966; Huang et al., 2008; Xi et al., 2014) and defined as the logMAR (log minimum angle of resolution) acuity associated with 75% correct identification (Xu et al., 2006; Zhou et al., 2006; Huang et al., 2009).

Contrast sensitivity (CS) was defined as the reciprocal of contrast threshold for detecting a grating with 79.4% accuracy. We measured CS using the quick CSF method (qCSF), which was recently developed by Lesmes et al. (2010) to accurately estimate CSF with greatly reduced testing times by sampling from pre-defined parameter space and updating the probability of CSF parameters based on subject’s performance. The stimulus space consisted of gratings contrasts ranging from 0.1% to 99% in steps of 1.5 dBs and spatial frequencies from 0.5 to 8 cycles per degree (cpd) in steps of 3 dBs. The qCSF’s parameter space is a four-dimensional grid of the four parameters that defined CSF, i.e., peak gain, peak frequency, bandwidth, and truncation level (Lesmes et al., 2010). The CSF curve was obtained after 100 qCSF trials. The area under contrast sensitivity function (AUCSF), a comprehensive measure of spatial vision over a wide range of spatial frequencies (van Gaalen et al., 2009; Lesmes et al., 2010), was calculated by integrating contrast sensitivity over spatial frequencies varying from 0.5 to 8 cpd. CSF in the upper right, upper left (trained location), and lower left visual field location of left eye (LE, trained eye), and the upper left of right eye (RE, untrained eye) was measured in four separate blocks and counterbalanced across subjects but held constant between pre- and post-training test sessions for a particular subject. Before pre-training CSF measurement, subjects practiced 20 trials to get familiar with the task.

### Training

Training was performed in the upper left visual field location of left eye and training spatial frequency was fixed at 5 cpd. A 3-down-1-up adaptive staircase procedure in which three consecutive correct responses resulted in a reduction of signal contrast (C_*n+*__1_ = 0.90C_*n*_), and one wrong response resulted in an increase in contrast (C_*n+*__1_ = 1.10C_*n*_) was used to control grating contrast (Levitt, 1971).

### EEG

The ten conditions conducted during pre- and post-training ERP measurements were summarized in Table 1. In the trained condition (spatial frequency: 5 cpd; retinal location: the upper left visual field location; trained eye: left eye), six different contrast levels were employed to obtain full CRF: 0, 4.26, 8.90, 18.61, 38.90, and 81.13% Michelson contrasts. These six conditions were randomly intermixed in four blocks, each consists of 300 trials. In the control conditions, EEG signals were recorded for gratings of 10 cpd and 38.9% contrast at the trained location (i.e., the upper left visual field location in the left eye with higher spatial frequency and a fixed contrast, Frequency change condition), gratings of 8.9% contrast at the upper right (Location change-contralateral condition), and the lower left location in the left (trained) eye (Location change-ipsilateral condition); and the upper left location in the right (untrained) eye (Eye change condition). These four control conditions were separately presented in four blocks of 200 trials each. Training and control conditions were counterbalanced across subjects.

**TABLE 1:** Stimulus details for ERP measurements.

Conditions	Eye	Location	Spatial frequency (cpd)	Contrast %
Trained	Left	Upper left	5	0
	Left	Upper left	5	4.26
	Left	Upper left	5	8.9
	Left	Upper left	5	18.61
	Left	Upper left	5	38.9
	Left	Upper left	5	81.13
Frequency change	Left	Upper left	***10***	***38.9***
Location change-contralateral	Left	***Upper Right***	5	***8.9***
Location change-ipsilateral	Left	***Lower Left***	5	***8.9***
Eye change	***Right***	Upper left	5	***8.9***

*Differences between the trained and each control condition are shown in italic bold.*

Scalp EEG data were recorded from 64 scalp electrodes (Neuroscan^®^) with an amplifier bandpass of DC to 100 Hz and a 60-Hz notch filter was digitized at 500 Hz. Vertical electro-oculogram (VEO) was recorded by electrodes placed above and below the left eye. Horizontal electro-oculogram (HEO) was recorded by electrodes placed at the outer canthus of the left and right eye. The reference electrode was placed on the top of the midline between electrodes C_*Z*_ and CP_*Z*_. Electrode impedance was kept <5 kΩ throughout recording.

EEG data were analyzed using EEGLAB (^1^; Delorme and Makeig, 2004) and ERPLAB (^2^; Lopez-Calderon and Luck, 2014) with home-made scripts. Signals were first referenced offline to the average of all the electrodes and filtered with a bandpass filter of 0.1–30 Hz. The data were then epoched starting at 200 ms before stimulus onset and ending 1000 ms after stimulus onset. The data exceeding ± 50 μV at electrode VEO and ± 15 μV at electrode HEO, or other activities exceeding ± 100 μV at any electrodes were excluded from analysis. The overall rejection rate was 17.27%. Remaining epochs were averaged according to the stimulus condition.

The peak amplitude was calculated with a moving window technique: the peak(s) within a certain time window was first determined for each subject and each condition (trained condition: 90–140 ms for P1, 160–300 ms for N1-P2 complex, 400–800 ms for N2; control condition: 110–160 ms for P1, 160–300 ms for N1-P2 complex, 400–800 ms for N2); then the peak value within a certain time window surrounding the first peak was derived for each subject and each condition (30 ms for P1 and 50 ms for N1-P2 complex and N2). To quantify the peak amplitude and latency of each component, the largest three electrodes among six contralateral posterior-occipital electrodes (P4, P6, P8, PO4, PO6, and PO8 in the right hemisphere and P3, P5, P7, PO3, PO5, and PO7 in the left hemisphere) were chosen for further analysis. Electrode sites were selected in temporo-parietal-occipital positions based on previous ERP studies of VPL (Ding et al., 2003; Song et al., 2005; Qu et al., 2010; An et al., 2012; Zhang et al., 2013; Itthipuripat et al., 2014, 2017; Garner et al., 2015; Ahmadi et al., 2018). The amplitude of each component was defined as the height of the peak in this average signal, and the latency was defined as its time to the peak. Amplitudes were measured as peak-to-peak voltages for N1-P2 complex rather than the base-to-peak amplitude due to uncertainties in establishing a baseline voltage for N1 and P2. For statistical analysis, amplitudes and latency were averaged across trials for each condition.

For the trained condition, we subtracted the ERP evoked by 0%-contrast stimulus from the ERP response evoked by all other contrasts to minimize the potential effects of anticipatory ERPs (Supplementary Figure 1).

### Statistical Analysis

The learning curve (i.e., log_10_ contrast sensitivity as a function of training session) was fitted with a linear function:

$${\text{log}}_{10}\,\mathrm{CS}\,\left(\text{session}\right)={\mathrm{CS}}_{0}+\alpha \times {\text{log}}_{10}\,\left(\mathrm{session}\right)$$

where *CS* denotes contrast sensitivity, *CS*_0_ is the intercept, and α is the slope of the learning curve (learning rate, or unit improvement at the trained condition).

To calculate the spatial frequency bandwidth of perceptual learning, we used the same methods as in our previous paper (Huang et al., 2008). Briefly, contrast sensitivity improvements of each observer were fit with a Gaussian function:

(1) $${\text{log}}_{10}\,\left[{\mathrm{CS}}_{\text{post}-\text{training}}\,\left(f\right)\right]-{\text{log}}_{10}\,\left[{\mathrm{CS}}_{\text{pre}-\text{training}}\,\left(f\right)\right]=a\,\text{exp}\,\left[-{\left(\frac{{\text{log}}_{2}\,\left(f\right)-{\text{log}}_{2}\,\left(f_{0}\right)}{\sigma }\right)}^{2}\right]$$

where *CS* denotes contrast sensitivity, *a* is the amplitude of the improvement, *f* is the spatial frequency, *f*_*o*_ is the spatial frequency with the maximum improvement, and σ is the standard deviation of the Gaussian function. The bandwidth (BW) of perceptual learning was defined as:

$$\mathrm{BW}=2\,\sqrt{\text{ln}\,2}\,\sigma$$

Standard deviations of all the estimated parameters were computed with a resampling method (Maloney, 1990).

The improvement of AUCSF, CS, and the amplitude of each ERP component was defined as:

$$I=\frac{{\text{Measure}}_{\text{post}-\text{training}}-{\text{Measure}}_{\text{pre}-\text{training}}}{{\text{Measure}}_{\text{pre}-\text{training}}}\times 100\%$$

The improvement of visual acuity (in logMAR) and latency of each ERP component was calculated as:

$$I=\frac{{\text{Measure}}_{\text{pre}-\text{training}}-{\text{Measure}}_{\text{post}-\text{training}}}{{\text{Measure}}_{\text{pre}-\text{training}}}\times 100\%$$

Pre- and post-training visual acuity, CS, BW, and learning improvement were compared using paired *t*-tests and corrected for multiple comparison based on FDR. Pre- and post-training latency and amplitude of each ERP component of control conditions were also compared using paired *t*-tests and corrected for multiple comparison based on FDR. Evidence against the null hypothesis was quantified using Bayes factors (*BF*_10_). Repeated ANOVA with Green house-Geisser correction was applied to the effects of training and contrast levels on the latency and amplitude of each ERP component of the trained condition.

### ERP Model Fitting

The Naka-Rushton equation was fitted to the ERP amplitude CRFs, i.e., amplitude of P1, N1-P2 complex, and N2 as functions of contrasts (Tolhurst et al., 1981; Li X. et al., 2008).

$$R\,\left(c\right)=b+R_{\text{max}}\,\frac{c^{s}}{c_{50}^{s}+c^{s}}$$

where *c* is the grating contrast, *b* is the baseline activity, *c*_50_ denotes the contrast at which the response reaches half of its maximum dynamic range, *s* is exponent controlling how quickly the CRF rises and reaches an asymptote, and *R*_*max*_ is the maximum response.

An inverted Naka-Rushton equation was fitted to the ERP latency CRFs, which was earlier shown to provide the best fit to the measured response latencies of neurons in striate cortex of cats and monkeys (Albrecht et al., 2002):

$$R\,\left(c\right)=L_{\text{max}}-R_{\text{shift}}\,\frac{c^{s}}{c_{50}^{s}+c^{s}}$$

where *c* is the grating contrast, *L*_*max*_ is the max latency, *c*_50_ denotes the contrast at which the latency reaches half of its minimum dynamic range, *s* is exponent controlling how quickly the CRF decreases and reaches an asymptote, and *R*_*shift*_ is the maximum reduction in latency.

Pre- and post-training model fitting parameters were also compared using paired *t*-tests and corrected for multiple comparison based on FDR. Evidence against the null hypothesis was quantified using Bayes factors (*BF*_10_). By systematically examining the best-fitting parameters of the Naka-Rushton equations to the amplitude and latency of different ERP components before and after training, we fulfilled the comparison between the contribution of early and late ERP components to perceptual learning within a unified theoretical framework.

## Results

### Behavioral Outcomes

#### Central Task

Subjects performed the central letter identification task with high accuracy during all the CSF measurements, training, and EEG sessions. There was no significant difference among the central letter identification performances in the four CSF tests before and after training (94.06, 97.12, 96.97, and 96.70% correct in pre-tests at the upper right, upper left, and lower left visual field location in the left eye (LE) and the upper left location in the right eye (RE) vs. 95.90, 93.77, 97.60, and 96.92% in post-tests, respectively; all *p* > 0.10). There was also no significant change in the central task performance during EEG measurements [pre-test: 93.15%, post-test: 94.61%, *t*(19) = 1.045, *p* = 0.31]. We concluded that the learning effects were not compensated from performance decrements in the central task.

#### Visual Acuity

**FIGURE 2:**
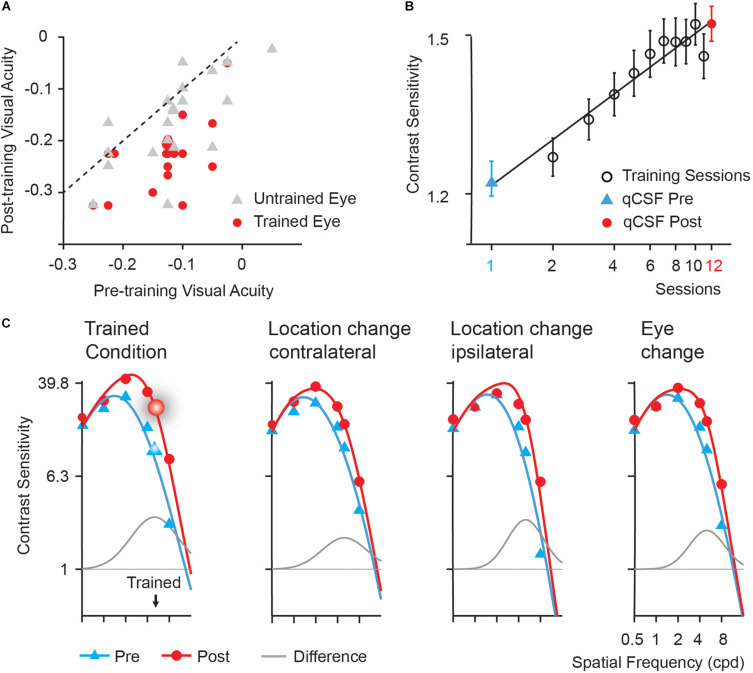
Behavioral results. **(A)** Post- versus pre-training logMAR visual acuity of trained (red circles) and untrained (gray triangles) eyes. Each symbol represents the data of one subject. The dashed line is the identity line (slope = 1), indicating no improvement. **(B)** Learning curve. Error bars represent standard errors across subjects. The first (blue triangles) and last data points (red circles) were derived from pre- and post-training CSF measurements in the trained condition, respectively. Black open circle: data from training phase. **(C)** Pre- (blue curves) and post-training (red curves) CSFs and the difference between the best fitting post- and pre-training CSFs (gray curves) measured in the trained location (the upper left), the upper right (Location change-contralateral) and the lower left (Location change-ipsilateral) visual field location in LE, and the upper left location in RE (Eye change). The enlarged symbols indicate the trained condition (spatial frequency: 5 cpd; location: the upper left; eye: LE) before (blue triangles) and after training (red circles). BW: the bandwidth of perceptual learning.

Training significantly improved visual acuity by 1.0 line in the left (trained) eye [from −0.13 to −0.23 logMAR, *t*(19) = 8.025, *p* < 0.005, *d* = 1.617] and 0.4 line in the right (untrained) eye [from −0.12 to −0.16 logMAR, *t*(19) = 3.320, *p* < 0.01, *d* = 0.582] after multiple comparison correction based on FDR (Benjamini and Yekutieli, 2001). The magnitude of improvement in the trained eye was significantly greater than that in the untrained eye [*t*(19) = 3.113, *p* = 0.006, *d* = 0.828]. In Figure 2A, we plotted visual acuity (logMAR) in the trained and untrained eyes after training versus that before training.

#### Learning Curve

Training at 5 cpd significantly improved CS by 38.17% [*t*(19) = 6.108, *p* < 0.001, *d* = 1.404]. Average learning curve was plotted in Figure 2B. The averaged best fitting curve has a slope of 0.306 log_10_ contrast sensitivity/log_10_ session (*r*^2^ = 0.887).

#### Contrast Sensitivity Functions

Contrast Sensitivity Functions (CSFs) measured in the upper left (trained location), the upper right, and the lower left location in LE (trained eye) and the upper left location in RE (untrained eye) of all the subjects before and after training were shown in Figure 2C. The AUCSF improved by 73.78, 53.35, 45.15, and 53.87% in the four conditions, respectively. The magnitude of AUCSF improvement in the trained location was significantly or marginally larger than that in the upper right [*t*(19) = 1.957, *p* = 0.065, *d* = 0.462], the lower left in LE [*t*(19) = 3.127, *p* < 0.05, *d* = 0.667], and the upper left in RE [*t*(19) = 1.987, *p* = 0.093, *d* = 0.479] after multiple comparison correction based on FDR. There was no significant difference among the magnitudes of improvement in the three control conditions (all *p* > 0.10, all *BF*_10_ < 4.20).

The spatial frequency bandwidth of perceptual learning indicates the generalization of training effect to other stimuli and tasks, were indexed by the full bandwidth at half height of the difference curve between the post- and pre-training CSFs, was 3.62 ± 1.96, 2.45 ± 1.62, 2.29 ± 1.19, and 3.55 ± 2.40 octaves (mean ± sd) for the upper left (trained location), the upper right, the lower left location in LE (trained eye), and the upper left in RE (untrained eye), respectively. The bandwidth of perceptual learning was significantly or marginally greater in the trained condition than in the upper right [*t*(19) = 2.177, *p* = 0.063, *d* = 0.643, paired *t*-test] and the lower left [*t*(19) = 2.877, *p* = 0.030, *d* = 0.778] in LE but not the upper left in RE [*t*(19) = 0.102, *p* = 0.920, *d* = 0.033] after multiple comparison correction based on FDR.

### ERP Outcomes

#### Overview

**FIGURE 3:**
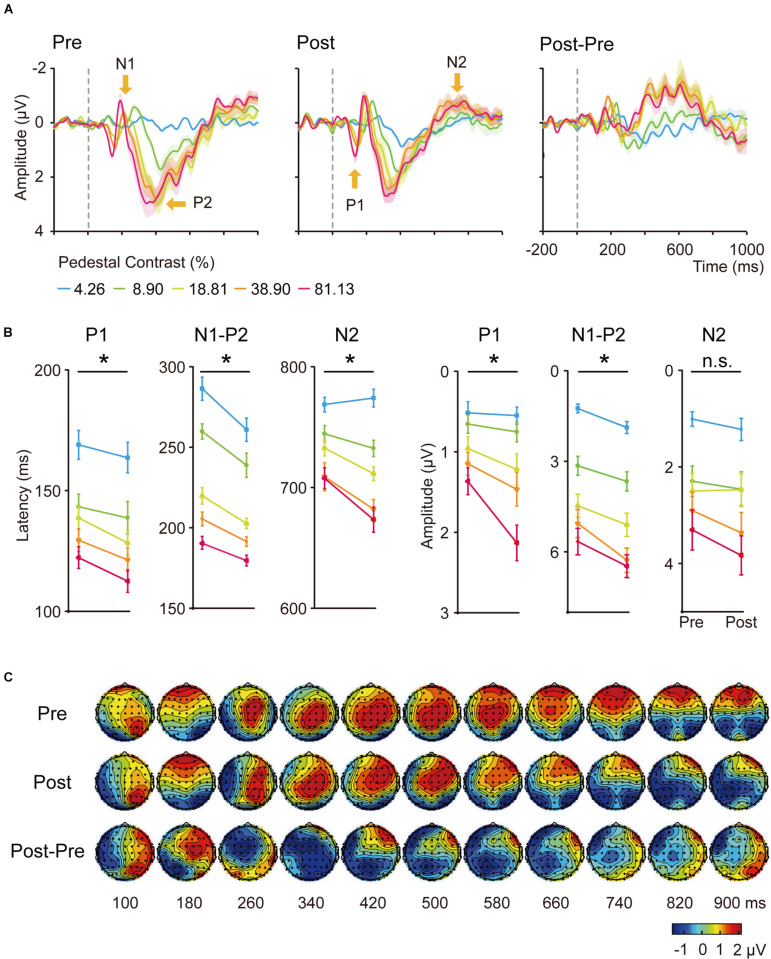
**(A)** Averaged ERP waveforms of the trained condition. The ERPs evoked by contralateral stimuli of 4.26, 8.90, 18.61, 38.90, and 81.13% Michelson contrast levels were subtracted by that evoked by contralateral 0%-contrast stimuli. Significant sensory ERP components, e.g., P1, N1, P2, and N2, were identified. Shaded regions denote standard errors across subjects. **(B)** Latency and amplitudes from early to late ERP components at each contrast levels of the trained condition in pre-training and post-training sessions. Statistical analysis showed that the latency and amplitude from early to late ERP components at each contrast levels were modified differently by training. Error bars represent standard errors across subjects. *: significant main effects of training; n.s.: non-significant. **(C)** The grand-mean topographical map series from 100 to 900 ms in steps of 80 ms evoked by stimuli of 81.13% contrast level of the trained condition in pre-training (upper part) and post-training (middle part) sessions. The difference topographical maps were also displayed (lower part). Four components occurred at this time window, from P1, N1, P2, to N2.

**FIGURE 4:**
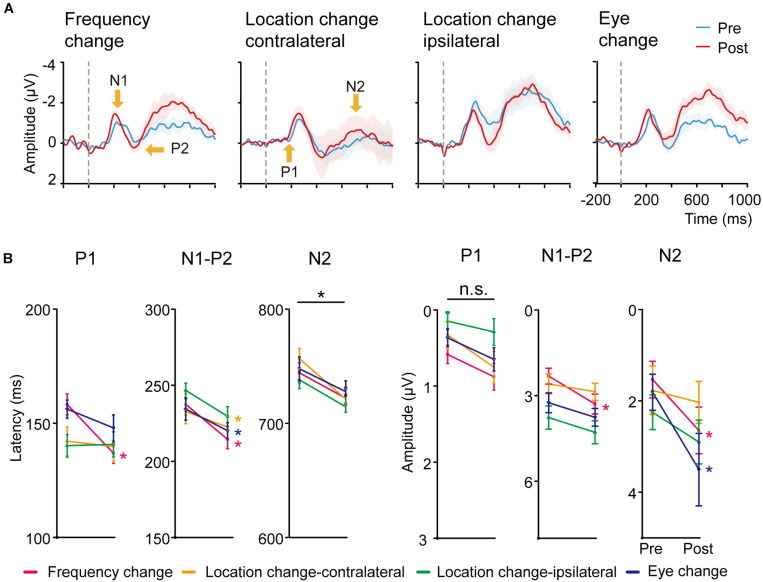
**(A)** Averaged ERP responses of control conditions. From left to right: Frequency change, Location change-contralateral (the upper right visual field location in LE), Location change-ipsilateral (the lower left location in LE) and Eye change condition (the upper left location in RE). Shaded regions denote standard errors across subjects. **(B)** Latency and amplitudes from early to late ERP components of the four control conditions in pre-training and post-training sessions. Statistical analysis showed that the amplitude and latency from early to late ERP components of the four control conditions were also modified differently by training. Error bars represent standard errors across subjects.

The grand average of stimulus-locked ERPs was shown in Figures 3, 4 for the trained and control conditions respectively. In the electrodes placed on the posterior-occipital cortex, we observed P1, N1, P2, and N2 components. The timing (Figures 3A, 4A) and topography (Figure 3C) of each ERP component were largely consistent with previous reports (Voorhis and Hillyard, 1977; Johnson, 1989; Duncan et al., 1994; Gonzalez et al., 1994; Woldorff et al., 1997; Luck et al., 2000; Vogel and Luck, 2000; Pernet et al., 2003; Potts, 2004; Key et al., 2005).

#### Trained Condition

Training was performed at 5 cpd in the upper left visual field location of LE. We first conducted six three-way ANOVA for the latency and amplitude of each of the following ERP component: P1, N1-P2 complex and N2, with hemisphere (left hemisphere vs. right hemisphere), training (pre-training vs. post-training), and contrasts levels (4.26, 8.90, 18.61, 38.90, and 81.13%) as within-subject factors. We found shorter latencies of P1 and N2 in the right hemisphere (contralateral) compared to the ones in the left (ipsilateral) hemisphere [*F*(1,19) = 5.290, *p* = 0.033, η*_*p*_^2^* = 0.218; *F*(1,19) = 144.013, *p* < 0.001, η*_*p*_^2^* = 0.883]. The amplitudes of P1, N1-P2 complex and N2 from the right hemisphere were larger than the ones in the left hemisphere [*F*(1,19) = 11.704, *p* = 0.003, η*_*p*_^2^* = 0.381; *F*(1,19) = 16.108, *p* < 0.001, η*_*p*_^2^* = 0.997; *F*(1,19) = 22.220, *p* < 0.001, η*_*p*_^2^* = 0.539].

Our further analyses focused on the contralateral (right) hemisphere. The latency and amplitude for each ERP component were then entered into a 2-way ANOVA with training (pre-training vs. post-training) and contrast levels (4.26, 8.90, 18.61, 38.90, and 81.13%) as two within-subject factors (Figure 3B). We found the latency of P1, N1-P2, and N2 components decreased significantly with contrast levels [*F*(4,64) = 31.723, 133.395, and 49.570, respectively, η*_*p*_^2^* = 0.625, 0.875, and 0.723, all *p* < 0.001) and training [*F*(1,19) = 6.128, 20.062, and 13.611, η*_*p*_^2^* = 0.244, 0.514, 0.417, respectively, all *p* < 0.05]. The interaction of the two factors was marginally significant for the latency of N2 component [*F*(4,76) = 2.729, *p* = 0.060, η*_*p*_^2^* = 0.126]. A follow-up simple effect test indicated the three higher contrast conditions reached significance for the latency of N2 component [*F*(1,19) = 0.30, *p* = 0.593, η*_*p*_^2^* = 0.016; *F*(1,19) = 2.52, *p* = 0.129, η*_*p*_^2^* = 0.117; *F*(1,19) = 6.23, *p* = 0.022, η*_*p*_^2^* = 0.247; *F*(1,19) = 7.11, *p* = 0.015, η*_*p*_^2^* = 0.272; *F*(1,19) = 9.08, *p* = 0.007, η*_*p*_^2^* = 0.323 for the five contrast levels separately].

The amplitudes increased significantly with contrast levels [*F*(4,76) = 21.692, 86.585, and 42.411, η*_*p*_^2^* = 0.533, 0.820, 0.691, for P1, N1-P2, and N2, respectively, all *p* < 0.001). Training also significantly increased the amplitude of P1 [*F*(1,19) = 6.085, *p* = 0.023, η*_*p*_^2^* = 0.243) and N1-P2 [*F*(1,19) = 16.521, *p* = 0.001, η*_*p*_^2^* = 0.465] but not N2 [*F*(1,19) = 0.463, *p* = 0.505). The interaction of the two factors was only significant for the amplitude of P1 component [*F*(4,76) = 3.607, *p* = 0.019, η*_*p*_^2^* = 0.160]. A follow-up simple effect test revealed that the amplitude of P1 component was only significantly increased when the stimulus contrast was 81.13% [*F*(1,19) = 21.39, *p* < 0.001, η*_*p*_^2^* = 0.530]. In sum, we observed shorter latency and increased amplitude for ERP components in response to stimuli presented at the trained location.

#### Control Conditions

We conducted paired *t*-tests (with multiple comparison correction based on FDR) for the latency and amplitude for each ERP component of the right hemisphere for Frequency change, Location change-ipsilateral, Eye change condition, and left hemisphere for Location change-contralateral condition (Figure 4B). For latency, training decreased P1 latency only at Frequency change condition [*t*(19) = 3.303, *p* < 0.01, *d* = 1.091] after multiple-comparison correction based on FDR. In contrast, significant or marginally significant reduction of latency was found for N1-P2 complex at Frequency change, Location change-contralateral, and Eye change-condition [*t*(19) = 3.629, *p* < 0.01, *d* = 0.803; *t*(19) = 3.138, *p* = 0.01, *d* = 0.675; *t*(19) = 2.144, *p* = 0.06, *d* = 0.504, respectively]; and for N2 component at all the four control conditions [*t*(19) = 2.125, *p* = 0.062, *d* = 0.630, *t*(19) = 3.668, *p* < 0.01, *d* = 0.944, *t*(19) = 2.991, *p* < 0.05, *d* = 0.719, *t*(19) = 1.893, *p* = 0.074, *d* = 0.497 for Frequency change, Location change-contralateral, Location change-ipsilateral, and Eye change condition, respectively] after multiple-comparison correction based on FDR.

For amplitudes, we found significant or marginally significant changes in N1-P2 complex amplitude for Frequency change condition [*t*(19) = 3.881, *p* < 0.005, *d* = 0.706] and in N2 amplitude for both Frequency change and Eye change condition (untrained) eye [*t*(19) = 2.990, *p* < 0.05, *d* = 0.549 and *t*(19) = 2.239, *p* = 0.074, *d* = 0.565] after multiple-comparison correction based on FDR.

The magnitude of improvement in N2 amplitude and latency at the trained condition was marginally larger than that in the Higher SF condition [*t*(19) = 1.782, *p* = 0.091, *d* = 0.25] and the Location change-contralateral condition [*t*(19) = 1.757, *p* = 0.095, *d* = 0.18], respectively. No significant difference of ERP changes in other control and corresponding trained conditions was found [*t*(19) = 1.582–0.082, *p* = 0.130–0.936, *BF*_10_ = 4.29–1.48].

### Model Analysis

**FIGURE 5:**
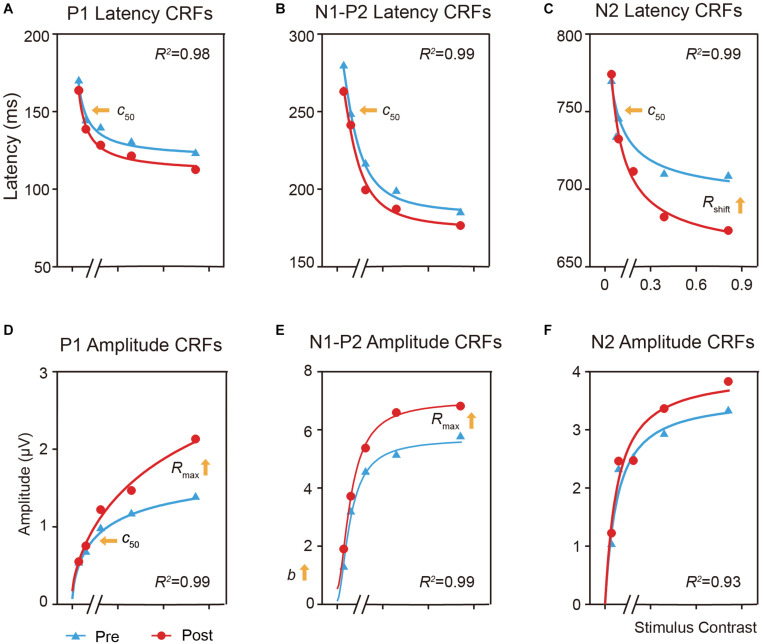
Effects of perceptual learning on the mean latency and amplitude of the P1, N1-P2 complex, and N2 components in the trained condition as function of contrasts (i.e., ERP-dependent CRF). **(A,D)** CRFs for P1 latency and amplitude. **(B,E)** CRFs for N1-P2 complex latency and amplitude. **(C,F)** CRFs for N2 latency and amplitude. For the latency CRF **(A–C)**, training lead to *c*_50_ improvement for both the P1 and N1-P2 complex, and *c*_50_ and response increase for N2. For the amplitude CRFs **(D–F)**, training led to *c*_50_ improvement and multiplicative response increase for P1, and multiplicative response and baseline increase for N1-P2 complex.

We plotted the mean latency and amplitude of the P1, N1-P2 complex and N2 components of the right hemisphere at the trained condition as functions of stimulus contrasts (i.e., CRF) and fitted with the Naka-Rushton equation (Figure 5; Tolhurst et al., 1981; Albrecht et al., 2002; Li X. et al., 2008).

For the latency CRF (Figures 5A–C), training increased the effective contrast (*c*_50_) by a factor of 0.72, or a decrease of 28% of its physical contrast, in the latency of P1 [*t*(19) = 2.925, *p* < 0.05, *d* = 0.624, multiple-comparison corrected based on FDR, Figure 5A] and by a factor of 0.70 in the N1-P2 complex [*t*(19) = 2.765, *p* < 0.05, *d* = 0.637, multiple comparison corrected based on FDR, Figure 5B]; and led to a shift of the contrast gain (*c*_50_) by a factor of 0.59 [*t*(19) = 4.179, *p* < 0.005, multiple-comparison correction based on FDR, *d* = 0.971] and a multiplicative response increase by a factor of 1.61 [*t*(19) = 2.090, *p* = 0.076, *d* = 0.446, multiple-comparison correction based on FDR] for N2 (Figure 5C).

For the amplitude CRF (Figures 5D–F), training led to a contrast gain (*c*_50_) improvement by a factor of 0.7 [*t*(19) = 2.673, *p* < 0.05, *d* = 0.472, multiple-comparison correction based on FDR] and a multiplicative response increase by 1.72 [*t*(19) = 3.713, *p* < 0.005, *d* = 0.490, multiple-comparison correction based on FDR] in the amplitude of P1 (Figure 5D); and a multiplicative response increase by a factor of 2.29 [*t*(19) = 2.160, *p* = 0.066, *d* = 0.381, multiple-comparison correction based on FDR] and baseline shift by a factor of 1.26 [*t*(19) = 2.576, *p* = 0.057, *d* = 0.517, multiple-comparison correction based on FDR] for the N1-P2 complex (Figure 5E). These results further showed that perceptual learning impacted neural processing differently across neural events at the trained condition.

## Discussion

In the present study, we tested the multi-stage hypothesis of perceptual learning. Behavioral results showed that training substantially improved visual acuity and CSFs, with the learning effect being particularly pronounced at the trained condition and partially transferred to control conditions. ERP results showed that training reduced the latency and increased the amplitudes on both early and late components for the trained condition. Further modeling analysis revealed a contrast-gain-related change in the latency of P1, N1-P2 complex, and N2, as well as response-gain-related changes in the latency of N2. Finally, for the untrained conditions, P1 showed reduced latency only at the high spatial frequency condition while N2 showed decreased latency for all control conditions.

The specificity of VPL has been the hallmark of perceptual learning and is often regarded as the evidence of a singular low-level process. In support of this hypothesis, fMRI studies revealed increased responses in the early retinotopic visual areas (Schwartz et al., 2003; Furmanski et al., 2004; Jehee et al., 2012). These results were further substantiated by EEG recordings showing post-training improvements in early visually evoked components over occipital electrode sites (Pourtois et al., 2008; Censor et al., 2009; Bao et al., 2010) and electrophysiological recordings in non-human primates linking behavioral performance with improvements in neuronal sensitivity in primary sensory areas (Ghose et al., 2002; Hua et al., 2010; Yan et al., 2014). In the current study, we observed contrast-dependent gain change both in the latency and amplitude of early P1 component, which resembles a previous single-unit study that recorded the responses of V1 neurons in cats and found that training increased neuronal contrast gain (Hua et al., 2010). P1 is a visually evoked exogenous response that reflected the encoding of sensory information in visual cortex (Voorhis and Hillyard, 1977; Gonzalez et al., 1994; Woldorff et al., 1997; O’Shea et al., 2010; Souza et al., 2013). Moreover, we found there is little improvement in the latency and amplitude of P1 at the location and eye change control conditions, which is also indicative of learning specificity. These findings were confirmed by the behavioral result of CSF measurements, i.e. magnitude of AUCSF improvement in the training location was larger than untrained conditions.

In contrast, there are also studies proposed that learning could be explained by selective reweighting of sensory information readout (Dorsher and Lu, 1998, 1999; Petrov et al., 2005; Liu et al., 2010), changes in attention and/or decision-making areas (Xiao et al., 2008; Zhang et al., 2010, 2013; Wang et al., 2012), or changes in both the sensory coding and the communication between the visual and the decision-making related areas (Chen et al., 2015, 2017). Interestingly, in our study, model-based analysis also revealed mechanisms of both response and baseline improvements at later stages. N1 reflects selective attention to basic stimulus characteristics and intentional discrimination processing (Näätänen et al., 1982; Luck et al., 2000; Vogel and Luck, 2000); P2 may reflect stimulus classification, and its amplitude increases with the stimulus complexity (Näätänen et al., 1982; Pernet et al., 2003; Crowley and Colrain, 2004; Potts, 2004); Late negative N2 has been associated with high-level decision-related processing and task demands (Johnson, 1989; Duncan et al., 1994; Mangun and Hillyard, 1995; Key et al., 2005). The response increment clearly indicates stimulus-independent effects of training on the latency of N1-P2 complex and N2 (Luck et al., 2000; Vogel and Luck, 2000; Pernet et al., 2003; Potts, 2004). The additive shift on the amplitude of N1-P2 complex ensures the effect that post-training responses will be higher than pre-training responses. These stimulus-independent improvements might reflect a top-down effect of training, such as decision and attention modulation on later stimulus processing stages. Also, we found significant improvement in the latency of N1-P2 complex and N2 at the spatial frequency, location, and eye transfer conditions. These results indicate unequal training effects across neuronal processing stages and the extent to which training transfers may depend on the specific stage of information processing. Using a motor training paradigm, Garner et al. (2015) also found transferability of training benefits was different across ERP components, i.e., N2 showed increased amplitudes and reduced latencies for both trained and untrained stimuli, while the onset of stimulus-locked lateralized readiness potential reduced only for the trained stimuli.

The multistage model could explain the existing divergent findings in perceptual learning (Sasaki et al., 2013; Shibata et al., 2016; Maniglia and Seitz, 2018). In accord with the multi-stage model, recent work in non-human primates found that V4 and the posterior inferior temporal (PIT) cortex both changed after training on an orientation discrimination task (Adab et al., 2014). Chen et al. (2015) has reported that training of a motion direction discrimination task is associated with changes in both V3A and connectivity between V3A and IPS. After training on a motion detection task, Shibata et al. (2016) found that the response changes in V3A were specific to the trained direction, independent of whether subjects performed the training task actively or only passively exposed to the stimuli, and significant response changes in V1 and the intraparietal sulcus (IPS) were found only when subjects performed the trained task on the trained motion stimulus, providing direct evidence for their two-plasticity model. Similarly, our results indicate different forms of experience-dependent plasticity: contrast-gain change in early P1 component and response gain/baseline increments in later N1-P2 complex and N2 component. Earlier ERP components might be more related to the physical properties (e.g., contrast) of stimuli which reflects the change of early sensory/feature processing stages, while later components were presumably modulated by top-down signals, which reflect the improvement in higher-level processing stages (Voorhis and Hillyard, 1977; Johnson, 1989; Duncan et al., 1994; Gonzalez et al., 1994; Woldorff et al., 1997; Luck et al., 2000; Vogel and Luck, 2000; Pernet et al., 2003; Potts, 2004; Key et al., 2005).

A recent ERP study also found significant changes in both early and late ERP components following training on a texture discrimination task (TDT) (Ahmadi et al., 2018). Specifically, they found a decrease in the C1 but not P1 amplitude, a decrease in both N1 amplitude and latency, and a significant increase in the P3 amplitude after training. In the current study, we found contrast detection training reduced the latency and increased the amplitude in both early and late ERP components, with different characteristics of contrast dependence and different underlying mechanisms explained within a quantitative modeling framework based on CRF measurements. The discrepancy was likely due to different training tasks and procedures used in the two studies. For example, Ahmadi et al. (2018) recorded ERPs while subjects performed the TDT at Session 1 and Session 2, with two full nights of sleep between sessions. Here we trained subjects on a contrast detection task for ten consecutive days and recorded ERPs during pre- and post-training tests.

We didn’t observe any significant C1 in our subjects. C1 is the earliest visual ERP component and is thought to be generated by neurons in V1 (Foxe and Simpson, 2002; Russo et al., 2003). There are three possibilities: (1) C1 is more vulnerable and difficult to be identified because of the specific orientation and folding of calcarine sulci of individual subjects (Kelly et al., 2008). (2) In order to measure ERP-based CRF, a high proportion of low contrast stimuli was involved in the current study (e.g., 0, 4.26, 8.90, 18.61, and 38.90% Michelson contrasts), which might not be able to elicit the subtle C1 effects or were overlaid by large individual differences in the functional anatomy of early visual cortex (Dougherty et al., 2003; Pourtois et al., 2008). (3) It usually needs more trials to isolate C1. We averaged over 200 trials, less than the previous studies (Ludwig and Skrandies, 2002; Russo et al., 2003; Bao et al., 2010). For example, Zhang et al. (2015) trained subjects with a similar peripheral (5° retinal eccentricity) grating orientation discrimination task and successfully isolated stimulus-related C1 epochs with a total of 450 ± 65 trials for each condition.

Although a large number of studies failed to detect latency change (Song et al., 2002, 2005; Pourtois et al., 2008; Bao et al., 2010; Qu et al., 2010; Wang et al., 2010; Hamamé et al., 2011; An et al., 2012; Zhang et al., 2013, 2015) and claimed that ERP amplitude instead of latency was more sensitive to training (Qu et al., 2010; An et al., 2012), a few studies reported training-induced ERP latency change (Skrandies and Fahle, 1994; Ludwig and Skrandies, 2002; Shoji and Skrandies, 2006; Garner et al., 2015; Diaz et al., 2017). In the current study, we found that the latency of both early and late components was shortened after training while increased amplitudes were seen in P1 and N1-P2 complex but not N2 component. The decrements of ERP latency might reflect improved efficiency of visual transmission from the lateral geniculate to higher cortical areas. In line with this claim, Mukai et al. (2007) found BOLD responses in putative attention-control areas reduced but the functional connectivity between frontoparietal areas and early visual cortex increased after training, indicative of improved processing efficiency following training. Note that we didn’t find changes in the amplitude of N2 components, which might be due to response saturation at this later stage.

In the current study, we found a mild improvement in visual acuity (e.g., 1.0 line in the trained eye and 0.4 lines in the untrained eye) following training on contrast detection. Visual acuity is usually thought to reflect the frequency limits of the visual system but the task, in fact, depends on a range of spatial frequencies, including low-to-medium spatial frequencies (Huang et al., 2007). Improvement of contrast sensitivity will likely benefit visual acuity, as evident in early studies with normal subjects (e.g., Zhou et al., 2007) as well as suffered population (e.g., Polat et al., 2004; Huang et al., 2008; Yan et al., 2015). Previous psychophysical studies have found that perceptual learning of contrast detection might decrease internal noise and/or finely tune perceptual template (Huang et al., 2009), with related brain area possibly down to LGN (Yu et al., 2016). In the current study, we found significant ERP changes in both early and late ERP components, which may reflect neuronal changes in both the representation stage and attentional processing (Voorhis and Hillyard, 1977; Luck et al., 2000; Fabiani et al., 2007). Relations among different studies that involved varied technological measures remains to be elucidated. One limitation of the current study is the lack of a control group that took pre- and post-training assessments (without training), which might weaken the interpretation of visual acuity improvement following training, although our focus was the improvement in contrast sensitivity and associated early and late ERP changes at the trained location following training and within-subject comparison between relative changes in trained and untrained conditions.

We also observed significant improvement in contrast sensitivity at the upper right, the lower left location in LE (trained eye), and the upper left location in RE (untrained eye). Our results were in general consistent with previous findings (Sowden et al., 2002; Yu et al., 2004; Casco et al., 2014), although there were differences in experimental settings. For example, in order to elicit a more reliable ERP response, we used a training frequency of 5 cpd, which is much lower than that in earlier studies (e.g., Zhou et al., 2007; Huang et al., 2008; Wu et al., 2020). Some have indicated greater improvement magnitude and transfer of perceptual learning was related to higher spatial frequencies (Wu et al., 2020). Another interesting finding is that training based on lateral masking could be more effective than protocols based on isolated Gabor stimuli to compensate for myopic vision (Camilleri et al., 2014). Future studies are needed to investigate whether a paradigm with higher spatial frequency training or lateral masking would result in better learning effects.

Taken together, our findings indicate that visual perceptual training leads to changes across different visual processing stages and the extent of learning and transfers may depend on the specific stage of information processing. Perceptual learning has been considered to be effective in improving deficient vision in clinical populations, e.g., amblyopia (Polat et al., 2004; Zhou et al., 2006), myopia (Durrie and McMinn, 2007; Yan et al., 2015), and presbyopia (Polat, 2009; DeLoss et al., 2015). On the other hand, many visual diseases demonstrated decreased amplitude and/or increased delay in both early and late ERP components (Levi and Harwerth, 1978; Sokol, 1983; Hess et al., 1985; Sengpiel and Blakemore, 1996; Koertvelyes et al., 2012). The current study, together with others (Skrandies and Fahle, 1994; Song et al., 2005; Shoji and Skrandies, 2006; Pourtois et al., 2008; Bao et al., 2010; Qu et al., 2010; Wang et al., 2010; Hamamé et al., 2011; Zhang et al., 2013, 2015), provided a more integrated way to understand visual rehabilitation and a potential method to modulate the efficacy of visual training (e.g., neuro-feedback, Saxby and Peniston, 1995; Hanslmayr et al., 2005; Vernon, 2005; Shibata et al., 2011; Zoefel et al., 2011). Another interesting open question is whether changes in both early and late ERP components happen concurrently or sequentially with training. Future studies should track brain activities during the course of training to give a full theoretical framework for understanding visual perceptual learning.

## Data Availability Statement

The raw data supporting the conclusions of this article will be made available by the authors, without undue reservation.

## Ethics Statement

The studies involving human participants were reviewed and approved by the Ethical Review Committee of Institute of Psychology, Chinese Academy of Sciences. The patients/participants provided their written informed consent to participate in this study.

## Author Contributions

JX, YZ, and C-BH designed the experiment. JX, PZ, and W-LJ collected the data. JX, JY, NC, G-TW, and C-BH conducted the analyses. JX, G-TW, YD, YZ, and C-BH wrote the manuscript. All authors contributed to the article and approved the submitted version.

## Conflict of Interest

The authors declare that the research was conducted in the absence of any commercial or financial relationships that could be construed as a potential conflict of interest.

## Footnotes

1 http://sccn.ucsd.edu/eeglab/

2 http://erpinfo.org/erplab/
