# Supplementary material for: Multi-Stage Cortical Plasticity Induced by Visual Contrast Learning
Source: Front Neurosci. 2020 Dec 21;14:555701. doi: 10.3389/fnins.2020.555701 (PMC7779615; doi:10.3389/fnins.2020.555701)
Supplement: Supplementary file 1 [file Data_Sheet_1.PDF]

Supplementary Materials 1

Captions to Figure.S1.

Figure S1. For the trained condition, we subtracted the ERP evoked by 0%-contrast stimulus from the ERP response evoked by all other contrasts to minimize the potential effects of anticipatory ERPs. In this case, the grand average ERP evoked by the post-training stimulus of 0% contrast (top grey line) was subtracted from that of 81.13% contrast (top red line), resulting in the subtracted ERP response (bottom red line).

Supplementary Materials 2

Matlab code for bandwidth calculation.

Main procedure.

```
%% This is to fit a set of two related DoG functions (DoG1 & DoG2) to two contrast sensitivity functions (CSF1 and CSF2).
%% The DoG functions was defined as: DoG1=a1*exp(-((x-b1)/c1)^2)-a2*exp(-((x-b2)/c2)^2); DoG2=DoG1+a3*exp(-((x-b3)/c3)^2).
%% In other words, an extra Gaussian function was used to capture the (potential) difference between CSF1 and CSF2 that
%% may arise from training, disease, drug treatment, and/or electrical stimulation. You may use with caution.
%% The range of the parameters may be modified based on your assumption.
%% You may also reduce the number of the parameters if you know what you are doing.
%% For example, statistical comparison between fits with and without the extra difference Gaussian (e.g. a3*exp(-((x-b3)/c3)^2))
%% will tell if there is significant difference between the two CSFs.
%% Jie & Chang-Bing @ IOP, CAS, 2020

clear all; clc;
CSF1=[0.0873 0.0356 0.0311 0.0585 0.0844];      % Pre-training contrast threshold data at 0.5 1 2 4 8 cycle per degree spatial frequency.
CSF2=[0.0661 0.0361 0.0143 0.0113 0.0152];      % Post-training data.
SF=[0.5 1 2 4 8];      % Spatial frequency (SF) in cycles per degree.
CSF1=log10(1./CSF1);      % Converted to log10 contrast sensitivity.
CSF2=log10(1./CSF2);
SF=log2(SF);      % Converted to log2 SF.
h_options=optimset('TolX',1e-12,'MaxIter',6000,'TolFun',1e-12);      % optimset Create/alter optimization OPTIONS structure.
lb=[0 0 0 0 0 0 0 0 0 ];    % Limit the maximum and minimum value of parameters.
ub=[100 100 100 32 32 32 100 100 100 ];
[para2,fval,exitflag,output]=fminsearch(@(parameters)bandwith_cost(parameters,SF,CSF1,CSF2),[1 0 1 1 1 1 1 1 1 1 1],lb,ub,h_options);
BandWidth(1)=2*sqrt(log(2))*para2(9);      % Compute bandwidth.
```

Define cost function in fitting DoG functions to CSFs

```
function minusL=bandwith_cost(para,x1,pre,post)
a1=para(1);
a2=para(2);
a3=para(3);
b1=para(4);
b2=para(5);
b3=para(6);
c1=para(7);
c2=para(8);
c3=para(9);
for i=1:size(x1,2)
```

```
    y1(i)=a1*exp(-((x1(i)-log2(b1))/c1)^2)-a2*exp(-((x1(i)-log2(b2))/c2)^2);
    y2(i)=a1*exp(-((x1(i)-log2(b1))/c1)^2)-a2*exp(-((x1(i)-log2(b2))/c2)^2)+a3*exp(-((x1(i)-log2(b3))/c3)^2);
end
minusL= sum((y1(:)-pre(:)).^2)+sum((y2(:)-post(:)).^2)
```
